# William John Penny

**Published:** 1905-03

**Authors:** 


### Obituary Notices.

### WILLIAM JOHN PENNY, F.R.C.S. ENG.,

*Consulting Surgeon to the Bristol General Hospital.*

WE regret to have to record the death of Mr. W. J. Penny, formerly residing in Clifton. He died near Mombasa, in British East Africa, on the 15th of December last. Mr. Penny was the eldest son of Mr. William Penny, of Crewkerne,

Somerset, and he received his professional education at King s College, London, where as a student he had a prosperous and brilliant career, popular alike with his fellows and teachers, as could be readily imagined by those who knew him in these early days and for some years after. He was an able and industrious student, and he had the good fortune to be at King's during the time that Professor (now Lord) Lister was actively carrying out and developing his great work of antiseptic surgery. Mr. Penny attracted the attention of Lord Lister by the skill and care with which he mastered the details of the antiseptic treatment of wounds: nothing could exceed the devotion and enthusiasm with which he followed the teachings of his great master. Mr. Penny held many appointments at King's College Hospital: he was house-surgeon, surgical registrar, and finally assistant-surgeon, when he seemed to have the prospect of a great professional future. At this period of his life Mr. Penny was a most genial companion, with many social qualities which gained him many friends. He possessed a decision of character and a firmness of purpose which made strangers immediately feel they were in the presence of a strong man to an extent which those who knew him at a later period would find it difficult to understand. Mr. Penny was rather above the middle height, had a good presence and a well-knit and strongly-built frame. He looked one of the most robust of men, but in spite of this he early broke down, and died in his 48th year.

As an athlete Mr. Penny was greatly distinguished in football, being captain of his Hospital XV. He played constantly for the United Hospitals and Somerset County, and on several occasions for England. He was considered by those who know of these matters to be the best "full back" of his time.

Locally Mr. Penny is best known for the good work he did for many years at the Bristol General Hospital. He was appointed house-surgeon to that institution in 1881, and held this office for the full period of three years to the satisfaction of all who were connected with the hospital. At the close of his term of office as house-surgeon he started in practice at Clifton. Soon afterwards he was elected assistant-surgeon, and in 1889 full surgeon; but after some seven years' work in this capacity he was compelled to resign from broken-down health, when he was placed on the consulting staff. There is no doubt that had his health remained good Mr. Penny would have had one of the largest surgical practices in the West of England. He was obliged to give up work at the early age of 37, when he retired to the country, and of late has been seldom seen by his old friends and colleagues. Mr. Penny was unmarried. He took the F.R.C.S. Eng. in 1885, was a member of the Bristol Medico-Chirurgical and other societies, and for a time was demonstrator of anatomy at University College, Bristol.

